# Natural Beauty

**Published:** 1892-09

**Authors:** E. L. S. Adams


### NATURAL BEAUTY.

One must learn to recognize beauty. Most eyes are untrained. A masterpiece of art is meaningless to the uncultivated eye. Half a lifetime may be spent in learning what to look for, to distinguish what is essential, what is characteristic, and to eliminate the rest.

The world, blinded by custom and prejudice and thirsting for novelty, ignores real or ideal beauty, satisfying itself with fashion, adhering to one new form until wearied, then thoughtlessly accepting another, only to sigh for a fresh change, and to laugh over the last caprice. Fashion is not beauty. Fashion is fleeting ; beauty is eternal, the same through all ages, The essential characteristics which make up beauty never change. Details may vary and be beautiful according to circumstances, but certain grand principles of art are fixed. Certain standards of beauty may be relied upon. One need not be swayed by the fancies of one person, nor by the superficial theories of another. Good taste is based upon the knowledge of these principles.

Beauty of the human form is to-day exactly what it was in ancient Greece ; it is the same through all the centuries, however blind we are to its characteristics through ignorance. The consensus of ages is a true verdict, and classic forms become safe models. Greek sculpture was wrought when the body received its highest cultivation, and was so beautiful as to be called divine.

This sculpture should be carefully and continuously studied, as well as pictures of good figures. They are to be made familiar that one may learn why they are good, why they deserve admiration. Most people fancy they admire these classic models, but it must be in imagination only, else why should they allow themselves to exemplify false standards of form, and positively distort their own God-given bodies ?

Searching for the highest standards of the human form, we discover that manly beauty and womanly beauty differ essentially. It is agreed that the type of manly proportion includes a comparatively large head, wide shoulders rather square, a torso tapering to the pelvis ; while the whole may be seven and a half heads in height, or an additional half head added to the length of the legs, giving a particularly elegant figure.

On the other hand, fine proportions for a woman are a small head, shoulders rather sloping and narrow, the torso full and widest at the hips; while the front line from the sternum over the abdomen should show first a gentle, and then a full outward curve.

The conventional figure of the day is at variance with this type. Every effort is made to imitate masculine characteristics. The shoulders are thrust up high and square, or made to appear so; the torso is made to taper in; and everything conceivable is done to make the waist look small. The front line is forced to take an inward curve below the bust, and the side lines, to form an awkward angle, in the hollow of which voluminous skirts are hung.

One should study sculpture with the new knowledge of these proportions most thoughtfully, till the rhythm of the lines has fastened itself upon the memory. Studying the pictures of the best artists of every age, we shall find these principles everywhere demonstrated.

The charm of womanly proportion is in the long curve from armpit to ankle, which is so different from the beauty of a manly figure. The depression at the so-called waist line—only the meeting of two large muscles, which in a beautiful woman should be slight—would better be ignored in the clothing, for the sake of the greater beauty of the whole sweep.

It is to be understood that the long curves are made up of shorter contours, one gently melting into another. A form made up of graceful sweeps alone would be a weak, nerveless, insipid thing.

These proportions should be so understood, and so thoroughly appreciated, as to be always in mind, else a beautiful human form will not be recognized. Use physical exercises to attain the perfection of these curves. Hang pictures showing them, where they may grow into your thoughts.

A knowledge of these primary truths will assist in the making of gowns. For instance, to preserve the beauty of the front line of a woman's figure the lesser beauty of the curve of the spine may be sacrificed. Any garment snug enough to touch the backbone down a good part of its length will press the soft front line out of shape.

An artist thoroughly in harmony with classic standards said a few days ago, "I have just seen a conventionally dressed woman who looked beautiful to me. Now what is the matter with me?"

That such a one should have received pleasure under the circumstances may be because conventional figures often have the same charm found in the symmetrical and elegant lines of ornament, or in those of a vase, a newel post, or a turret. There is a desirable elegance in length or height. Slender figures may attain this excellence. The lines of a stout figure may even impart a satisfaction allied to that which we take in the form of a jug or a bottle.

All this may be pleasing or otherwise, but such an effect is not human. Smooth, sheathlike garments suggest the enamel or glaze of pottery. They may be beautiful in color and texture at the same time that they are wholly unsuited to the undulating motion of living beings. They are in every sense opposed to the necessary freedom of bone and muscle. The soul needs the freest, most elastic environment to encourage its full expression. Every means should be at hand to facilitate that expression, every avenue opened, every stiff, inflexible restraint removed, every intrusive restriction put out of the way.—*E. L. S. Adams, in Harper's Bazar.*

